# Identification and characterization of histone lysine methylation modifiers in *Fragaria vesca*

**DOI:** 10.1038/srep23581

**Published:** 2016-04-06

**Authors:** Tingting Gu, Yuhui Han, Ruirui Huang, Richard J. McAvoy, Yi Li

**Affiliations:** 1 State Key Laboratory of Plant Genetics and Germplasm Enhancement and College of Horticulture, Nanjing Agricultural University, Nanjing, P. R. China; 2 Department of Plant Science and Landscape Architecture, University of Connecticut, CT 06269, USA.

## Abstract

The diploid woodland strawberry (*Fragaria vesca*) is an important model for fruit crops because of several unique characteristics including the small genome size, an ethylene-independent fruit ripening process, and fruit flesh derived from receptacle tissues rather than the ovary wall which is more typical of fruiting plants. Histone methylation is an important factor in gene regulation in higher plants but little is known about its roles in fruit development. We have identified 45 SET methyltransferase, 22 JmjC demethylase and 4 LSD demethylase genes in *F. vesca*. The analysis of these histone modifiers in eight plant species supports the clustering of those genes into major classes consistent with their functions. We also provide evidence that whole genome duplication and dispersed duplications via retrotransposons may have played pivotal roles in the expansion of histone modifier genes in *F. vesca*. Furthermore, transcriptome data demonstrated that expression of some SET genes increase as the fruit develops and peaks at the turning stage. Meanwhile, we have observed that expression of those SET genes responds to cold and heat stresses. Our results indicate that regulation of histone methylation may play a critical role in fruit development as well as responses to abiotic stresses in strawberry.

Chromatin is a complex composed of histones, chromosomal proteins, DNAs and small RNAs. The nucleosome is the basic unit of chromatin, consisting of a histone octamer (two of each histone 2A, histone 2B, histone 3 and histone 4), surrounded by a 146–148 bp DNA wrapping. The post-transcriptional modifications of the N-terminal tails of core histones could affect the nucleosome spacing, higher-order nucleosome interaction, and greatly affect the accessibility of the transcriptional regulatory proteins. Thus, in eukaryotes, post-transcriptional histone modifications are a determinant of the active/silent state of the associated genes, and are of great importance to a variety of important biological processes^1^.

The covalent modifications of histones include methylation, acetylation, phosphorylation, ubiquitation and SUMOylation^2^. Methylation on the lysine residues is among the most variant and important histone modifications. The majority of methyl marks are located on lysine 4, 9, 27, 36, 79 on histone H3, and lysine 20 on histone H4. The different active/silent chromatin states are characterized by different combinatorial patterns of histone modifications. Generally speaking, methylations on H3K9, H3K27, H3K79 and H4K20 are silent marks, while methylations on H3K4 and H3K36 are active marks^3^,^4^. Although having some variations, the mechanisms determining the chromatin states are quite conserved in plants and animals.

Histone lysine methylation is dynamic during organ development, and determined by histone modifying proteins including histone lysine methyltransferases (HKMTases), histone demethylases (HDMases) and histone turnovers. The majority of HKMTases have a SET (*S*uppressor of variegation, *E*nhancer of *zeste* and *T*rithorax in *Drosophila*) domain mediating the methyltransferase catalytic activity^5^. The only known HKMTase that does not have a SET domain is Dot1/Dot1L, which is responsible for H3K79 methylation^6^,^7^.

Based on the amino acid sequence conservation of SET domains, there are seven classes of SET protein encoding genes in *Arabidopsis*, each of which have preferred targets^5^,^8^. Class I consists of three polycomb group genes homologous to the *Drosophila* ortholog *E(Z)* (*E*nhancer of *Zeste*), capable of transferring methyl groups to H3K27. Class II consists of five SET genes homologous to the *Drosophila* ortholog *ASH1*, responsible for methylations on H3K4 and/or H3K36. Class III is another group of SET genes responsible for the active mark H3K4me1/2/3, similar to the *Drosophila* ortholog *TRX*. Class IV is plant-specific, responsible for monomethylation on H3K27, a silent mark essential for transposon silencing^9^. Class V is the largest SET group consisting of 15 SET genes homologous to *Drosophila SU(VAR)3–9*. Similar to their orthologs in animals, class V SET genes in *Arabidopsis* play an essential role in the establishment of heterochromatic mark H3K9me1/2/3, as well as H4K20me and H3K27me2. Class VI and VII consist of genes having a SET domain with functions to be determined.

Histone HDMases consist of two major types of enzymes, the LSD (*L*ysine *S*pecific *demethylase*) type and the JmjC domain-containing HDMases^10^. LSD HDMases can only demethylate mono- and di-methylated lysine residues^7^. LSD family in *Arabidopsis* has four members, being able to demethylate H3K4me1/2 and H3K9me1/2. While JmjC is a much larger gene family consisting of 21 genes in *Arabidopsis*, and is compatible with trimethylated residues^11^. Within the JmjC family, members with the activity to demethylate methyl groups on H3K4, H3K9, H3K27, H3K36 and H4K20 have been identified^7^. H3K79 methylation is mediated by the only non-SET domain HKMTase Dot1/Dot1L, thus it is tempting to speculate that another class of histone HDMase might be responsible for H3K79 demethylation.

The first plant SET genes identified are *CURLY LEAF* (*CLF*) and *MEDEA* (*MEA*) in *Arabidopsis thaliana*^12^,^13^. Since then, the identification and functional investigation of histone HKMTases and HDMases in plants have been the subject of numerous studies. These studies suggest that HKMTases and HDMases are pivotal in phase transitions between sporophyte and gametophyte, gametophyte and seed development, embryo-seedling transition, induction of flowering, and vernalization^1^. In addition, histone methylations determined by both HKMTases and HDMases play an important role in the memory mechanism in response to recurring stresses^1^,^14^. For example, the “memory genes” responded to recurrent dehydrations maintain the active mark H3K4me3 during the recovery phase when transcription is low, which serves as a mark of “transcriptional stress memory”^14^. The list of cellular processes known to involve HKMTases/HDMases is still growing, and these histone modifiers are believed to play essential roles in all aspects of regulations of plant development.

In spite of the essential roles of histone modifications in cellular processes, little is known about histone modifiers in strawberry. The cultivated strawberry (*F*. x *ananassa*) is a young crop species as a model plant considered to be non-climacteric. *F*. x *ananassa* has an extremely complex octaploid genome harboring 56 chromosomes (2*n* = 8*x* = 56) derived from 4 diploid ancestors. Thus, the sequenced diploid woodland strawberry *Fragaria vesca* with a small genome (240 Mb, 2*n* = 2*x* = 14) offers substantial advantages for genomic research^15^. In this study, we identified genes encoding the histone lysine methylation modifiers, both HKMTases and HDMases in *F. vesca*. Comprehensive studies about the phylogeny, evolutionary history, structure, expression patterns in different stages/organs and in response to abiotic stresses were performed to give an overview of this important group of genes in *F. vesca*. This study provides the first characterization of the full set of histone lysine methylation modifiers in strawberry, and should greatly facilitate the functional characterization of those epigenetic regulators in this economically important crop species.

## Results

### Identification of genes encoding putative histone HKMTases containing SET domains in *F. vesca*

**Figure 1:**
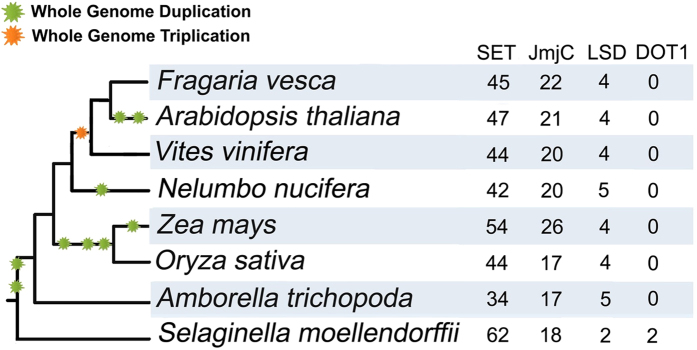
The Taxonomy Common Tree of the eight species (*F. vesca*, *A. thaliana*, *V. vinifera*, *N. nucifera*, *O. sativa*, *Z. mays*, *A. trichopoda* and *S. moellendorffii*) and the numbers of SET, JmjC, LSD and Dot1/Dot1L genes retained in each genome. The Taxonomy Common Tree was constructed online by Taxonomy Browser in NCBI (http://www.ncbi.nlm.nih.gov/Taxonomy/CommonTree/wwwcmt.cgi).

To identify histone HKMTases, the full alignment of SET domains downloaded from Pfam was searched against the *F. vesca* proteome by HMMER toolset (Methods for details). This sequence-based search identified 45 SET domain-containing genes in *F. vesca* (Fig. 1, Table 1 and Supplementary Table S1). To better understand the expansion and evolutionary history of SET genes in *F. vesca*, genes encoding SET-domain containing proteins were also identified in seven other species representing the major clades of terrestrial plants (Fig. 1). The basal angiosperm *Amborella trichopoda* is suggested to be the single living representative of the sister lineage to all other extant flowering plants^16^*. A. trichopoda* originated prior to the split of eudicots and monocots, and has not experienced any whole genome duplication (WGD) since then^16^; while the other seven angiosperms had several rounds of whole genome duplication/triplications after their split from *A.trichopoda* (Fig. 1, data from PGDD website, http://chibba.agtec.uga.edu/duplication/)^17^, which should have contributed to the evolution of SET genes. To standardize gene names, the *Arabidopsis* genes with known functions were named as published, following the standard gene symbol conventions with all capital letters; while genes in other species were named as *SET1-62* following the species abbreviation.

### Expansion and evolution of SET genes in *F. vesca*

**Figure 2:**
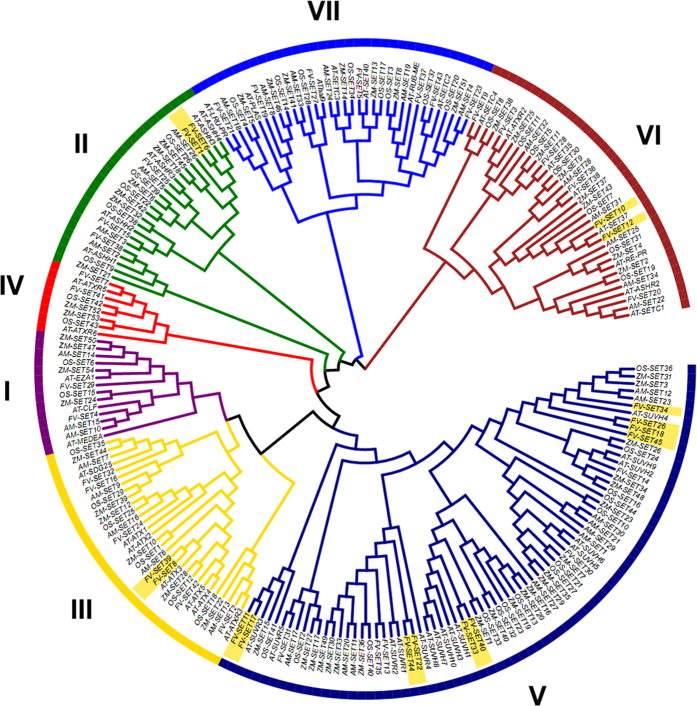
A most likelihood phylogenetic tree of predicted SET genes identified *F. vesca*, *A. thaliana*, *O. sativa*, *Z. mays* and *A. trichopoda*. The phylogenetic tree was constructed based on the amino acids sequences of the SET domains with 1000 bootstrapping replicates. The *F. vesca* lineage-specific duplicated gene pairs (since its split form *A. thaliana*) are highlighted as yellow. The seven classes of SET genes are marked by different colors. Refer to Table 1 for more basic information of SET genes in *F. vesca* and *A. thaliana*.

In order to investigate the classification of *F. vesca* SET genes, phylogenetic trees were constructed using the Maximum Likelihood method (see Methods for details) based on amino acid sequences in the conserved SET domains on the SET genes identified in the agiosperms *F. vesca*, *A. thaliana*, *Z. mays*, *O. sativa* and *A. trichopoda* (Fig. 2). It is noted that the topologies of phylogenetic trees constructed by different methods are slightly different, and the results of the bootstrapping analysis for some nodes are lower than 60 (Fig. 2, Supplementary Fig. S1, S2–S6). Thus, domain composition for the whole proteins (Supplementary Fig. S1) and motif construct for the SET domains (Supplementary Fig. S2) were taken into consideration for the classification of SET genes as well. Accordingly, the SET genes could be grouped into seven classes (Fig. 2 and Table 1), which is consistent with previously reported in *A. thaliana*^8^. Class I–V consist of most of the canonical SET genes known to be involved in the catalysis of histone methylation (in *Arabidopsis*); while class VI and VII consist of relatively shorter genes with no known specificity (Table 1 and Fig. 2).

The seven SET classes have different domain architectures and motif compositions (Supplementary Fig. S1–S6). In addition to the domain compositions identified by pfam, the 20 most common motifs embedded in the SET domains were identified by MEME for class I–V SET genes (Supplementary Fig. S2). Class I has few domains beside SET, but all the SET domains in this class consistently have the class I-specific motif 16 and 17. Although four SET genes in other classes have the motif 16 as well, the sequences are highly degenerate. Class II is characterized by AWS, SET and Post-SET domains, motif 1, 2, 3, 4, 9, and a class II-specific motif 20. Class III SET genes have PHD, zf-HC5HC2H_2, SET and Post-SET domains at high frequency, motif 1, 2, 3, 9 and class III-specific motif 7, 8, 12 and 15. Class IV is characterized by PHD and SET domains, and motif 1, 2 and class IV-specific motif 18. Class V SET genes have SAD_SRA, Pre-SET, SET and Post-SET domains, motif 1, 2, 3 and 4 at high frequency, and several class V-specific motif 5, 6, 11, 12, 14 and 15 (Supplementary Fig. S1,S2). While class VI and VII SET genes are relative short, and have few domains known to be essential for an HKMTase catalysis function.

**Figure 3:**
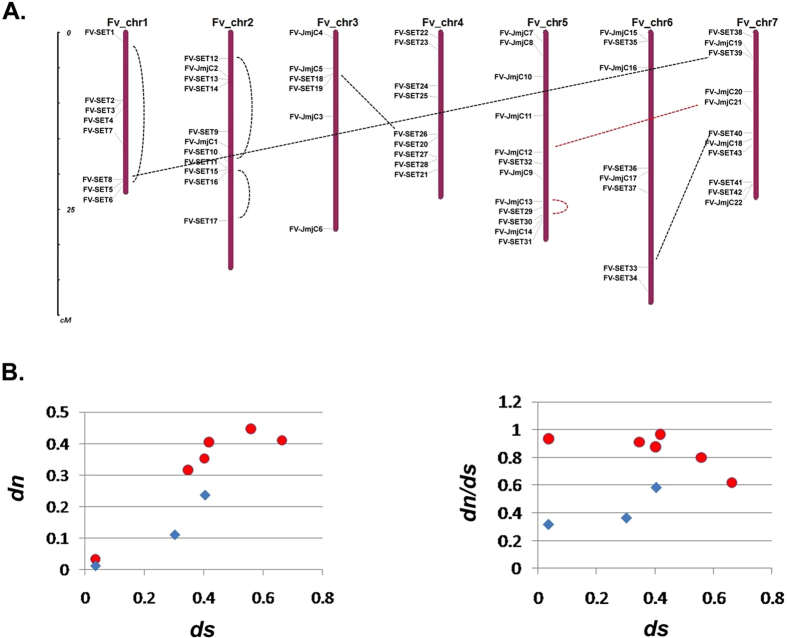
*F. vesca* lineage-specific duplicated SET gene pairs in class V exhibits stronger positive selection than in other SET classes. (**A**) Chromosomal locations of SET and JmjC genes on the seven chromosomes of *F. vesca*. The lineage-specific duplicated SET and JmjC pairs are connected by dashed lines. The scale on the left is in megabases. (**B**) The correlation between *ds vs. dn*, and *ds vs. dn/ds* for those duplicated gene pairs. Red circles denote duplicate genes pairs in class V, while blue diamonds denote those in other classes. For the *FV-SET34/45/18/26* gene set, *ds* and *dn* were calculated for *FV-SET34 vs.45, 34 vs.18 and 34 vs.26*, according to the phylogenetic tree shown in Supplementary Fig. S5.

To explore the detailed evolutionary history of the SET domain containing genes, the phylogenies of each class were investigated. In general, most of the SET genes reside in the sub-clades consisting of genes from all five species (Supplementary Fig. S3–S6), but there are some exceptions as well. For example, the *AT-RE-PR* sub-clade in class VI, and the *AT-SETC3* sub-clade in class VII do not have an *F. vesca* member, indicating a lineage-specific loss in *F. vesca* (Supplementary Fig. S5,S6). On the other hand, in some sub-clades, two *F. vesca* genes clustered together with either a single *Arabidopsis* gene, or without any corresponding genes in *Arabidopsis* (Fig. 2, highlighted by yellow), indicating a lineage-specific duplication in *F. vesca* (duplications happened in *F. vesca* after its split from *A. thaliana*). In total, there are 7 pairs of such *F. vesca* SET genes, with 4 pairs in class V and the other 3 in other classes. To study the evolutionary constraints performed on the 7 recent duplicates in *F. vesca*, *dn* (nonsynonymous substitutions per site) and *ds* (synonymous substitutions per site) between each duplicate were calculated. *dn* of class V duplicated pairs are higher than other classes (Fig. 3B). Furthermore, *dn/ds* value of class V duplicates ranges from 0.62 to 0.93, significantly higher than the 3 duplicates from other classes (Fig. 3B), indicating a relaxation of negative selection on the class V duplicated pairs after duplication events happened^18^. In addition, some of those recent duplicates are coupled with domain gains and losses. For example, in duplicate pair *FV-SET22* and *FV-SET44* of class V, *FV-SET22* obtained two domains at the N-terminal (Supplementary Fig. S3). Overall, recent duplicate pairs were more frequently originated or maintained in class V, and class V SET gene duplicate pairs evolved faster than other classes.

On the DNA level, WGD, large segmental duplication, or tandem duplication might lead to those duplicated pairs. To evaluate the relative contribution of those mechanisms in the expansion of the SET gene family in *F. vesca*, all SET genes were mapped to the seven chromosomes (Fig. 3A) and analyzed by MCscan^19^. The MCscan results suggest that in *F. vesca*, 6 out of the 45 SET genes were related to WGD, while the others resulted from dispersed duplications (Supplementary Table S2,S3). In class V, *FV-SET34/45/18/26* cluster with a single *Arabidopsis* gene *AT-SUVH4* (Fig. 2), indicating that more than one duplication event happened to the *F. vesca* orthologs of *AT-SUVH4*. The fact that both *FV-SET18* and *FV-SET* 26 have a single exon suggests that retro-transposition may have contributed to the expansion of this gene set. In summary, our results suggest that most of the *F. vesca* SET genes originated before the split of eudicots and monocots, and that WGDs, dispersed duplications via retro-transpositions in some cases, have contributed to the evolution of SET genes in *F. vesca* as well.

### Identification of genes encoding histone HDMases and investigation of their evolution in *F. vesca*

To investigate the histone HDMases in *F. vesca*, LSD HDMases and JmjC domain-containing HDMases were identified by sequence-based search using HMMER toolset. All the LSD HDMases characterized previously contain two domains, a SWIRM domain and an amino oxidase domain^7^. Thus, the proteins with both domains were identified as putative LSD HDMases.

**Figure 4:**
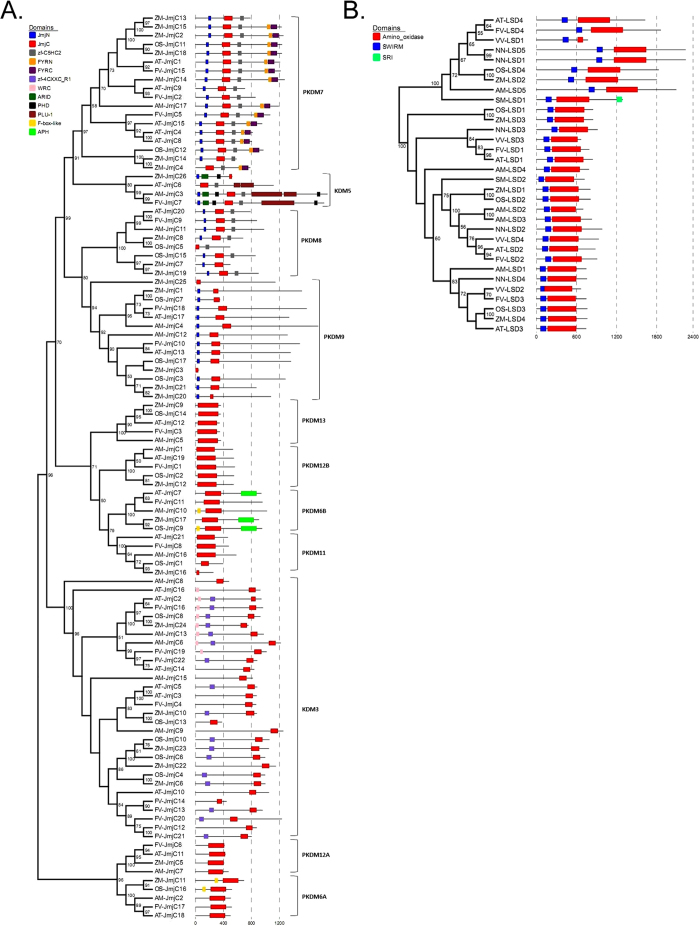
Most likelihood phylogenetic trees and schematic diagrams for domain composition of JmjC (**A**) and LSD (**B**) genes in the species investigated. The phylogenetic tree was constructed based on the amino acids sequences of either the JmjC domain (**A**) or the whole LSD protein (**B**) with 1000 bootstrapping replicates, and the results of the bootstrapping analysis larger than 50% are shown.

In total, the sequence-based search identified 5, 4, 4, 4 and 4 genes encoding proteins with both the SWIRM and amino oxidase domains in *A. trichopoda, O. sativa*, *Z. mays, A. thaliana* and *F. vesca*, respectively (Fig. 4, Table 2 and Supplementary Table S4). The consistent number of LSD HDMases indicates that duplication events may not contribute much to the evolution of LSD HDMases in angiosperms.

In contrast to LSD HDMases, the number of JmjC HDMases varies in the five species. In total, *A. trichopoda, O. sativa*, *Z. mays, A. thaliana* and *F. vesca* have 17, 17, 26, 21 and 22 JmjC domain-containing genes respectively (Fig. 1 and Fig. 4). According to phylogenetic trees and domain constructs, JmjC HDMases are grouped into 9 classes (Fig. 4), which is consistent with previous reports^20^. Specifically, *F. vesca* lineage-specific duplications only happened in the KDM3 class, in which the sister group of *AT-KDM3C* (*AT*_*JmjC10*) in *F. vesca* has five members (*FV*_*JmjC12, 13, 14, 20* and *21*). Interestingly, among all the JmjC domain-containing genes, *FV*_*jmjC12* and *FV*_*jmjC21* are the only two genes having a single exon (Supplementary Fig. S7), suggesting that the ancestor of *FV*_*JmjC12* and *FV*_*JmjC21* resulted from a retrotransposition event where transcribed messenger RNA was inserted into the genome to form the ancestor of *FV*_*JmjC12* and *FV*_*JmjC21*. In most classes, the *F. vesca* JmjC HDMases have not expanded, but in the KDM3 class, a series of duplication events occurred leading to the *F. vesca* lineage-specific expansion in this particular class.

In order to investigate which mechanisms might have contributed to those duplication events, JmjC genes were mapped to *F. vesca* chromosomes (Fig. 2) and analyzed by MCscan. MCScan results suggest that out of the 26 HDMase genes, 2 were WGD-related, and 24 resulted from dispersed duplications (Supplementary Table S2,S3). Therefore, similar to SET HKMTases, most of the *F. vesca* LSD and JmjC HDMases originated before the split of eudicots and monocots; and recent dispersed duplications and retro-transpositions might have played a pivotal role in the evolution of histone HDMases in *F. vesca*.

### Expression profiles of histone HKMTases and HDMases in flower and fruit development in *F. vesca*

To investigate the expression profiles of individual histone HKMTase and HDMase genes in different organs and developmental stages, transcriptome data were investigated in flower development and early-stage fruit development^21^,^22^. One out of 45 *F. vesca* SET genes have no expression data available from the database, and were omitted from the following analysis.

**Figure 5:**
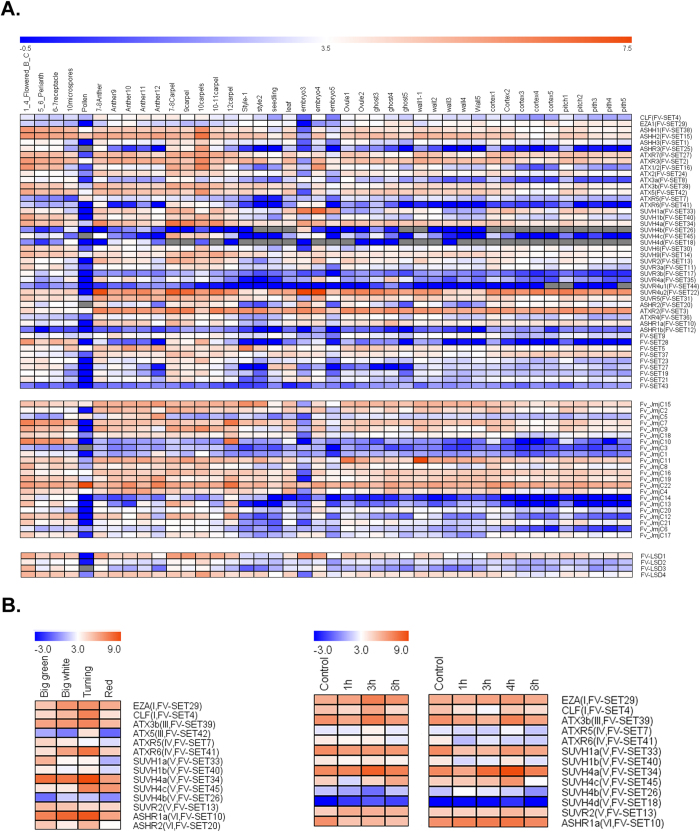
Expression profiles of identified histone HKMTase and HDMase genes in *F. vesca*. (**A**) The mRNA levels of histone modifiers in different tissues in flower and early-stage fruit development. The expression levels (RPKM) for the genes of interest were directly retrieved from http://bioinformatics.towson.edu/strawberry/) and plotted in log2 scale. (**B**) Expression profiles of SET genes in flesh (including pith and cortex, without seeds) during fruit ripening. (**C**) Expression profiles of SET genes in response to heat and cold stresses. For (**B**,**C**), the expression levels relative to GAPDH were measured by quantitative RT-PCR, and displayed in log2 scale. Three biological replicates and three technical replicates were done for each data point.

The genes encoding HKMTases and HDMases have quite diversified expression patterns (Fig. 5A). Firstly, for the seven classes of SET genes, the members within a particular class show different tissue/stage-specific expressions. For example, in class I, *CLF* is moderately expressed in each tissue/developmental stage; while the mRNA of *EZA1* is depleted in pollen and the early-stage embryo, and is more enriched in the developing pith and cortical tissues of strawberry flesh. Secondly, the respective members of the recently duplicated SET pairs have different tissue-specificity. Based on available transcription data, five out of the six duplicated *F. vesca* SET pairs with transcription data available have a similar expression pattern: one gene of the duplicated pair is more evenly and ubiquitously expressed, while the other gene is silent in the flesh (pith and cortex), the anther, and in some tissues in the seed (embryo, ghost, wall). It suggests that although highly conserved on amino acids sequence, those duplicated genes are differently regulated. Thirdly, LSD and different classes of JmjC HDMases show different expression profiles in different organs/stages as well; and the recent JmjC HDMase duplicates also express differentially (Fig. 4). Overall, the expression of histone HKMTase and HDMase genes has different organ/stage specificity, indicating a functional diversification coupled with the expansion of those gene families in *F. vesca*.

On the other hand, the different organs/stages have very different combinatorial expression patterns of HKMTase and HDMase genes (Fig. 5A). Firstly, most of the SET genes are expressed at extremely low level in pollen, with the majority of the class V and class I SET genes silent there. Secondly, the mRNAs of all the LSD HDMases and most of the JmjC HDMases are depleted in pollen as well, but interestingly, *FV-JmjC5* and *FV-JmjC16* show highest expression, indicating that those two JmjC HDMases might play a dominant role in histone demethylation in pollen. Thirdly, in the developing strawberry flesh (pith and cortex), both active and silent mark-related SET genes, LSD and JmjC genes show decreasing expression levels during early-stage fruit development (pollination to big green). Fourthly, overall, genes encoding both HKMTases and HDMases express higher in tissues of developing flowers (carpels, perianth, flowers, receptacles and microspores) than in other tissues. Thus, the different tissues in different developmental stages have diversified expression patterns of histone lysine methylation related genes, indicating the specific regulatory roles of those genes in cellular processes.

The expression profiles shown above reveal that duplicated SET pairs are quite distinct, with one silent in early-stage fruit development, while the other relatively ubiquitously expressed in all tissues, (Fig. 5A). To investigate how those duplicated SET genes express during strawberry fruit ripening, the expression levels of the more ubiquitously expressed genes were investigated in strawberry fleshy fruits (stripped with seeds, including pith and cortex only) at big green stage, big white stage (with red seeds and white flesh), turning stage (with red seeds and light white flesh) and red stage (2–3 days after turning stage) by quantitative RT-PCR assays (Fig. 5B). In addition, a subset of SET genes representing different classes was investigated as well. In contrast to the overall decreasing expression during early-stage fruit development, the mRNA levels of a substantial number of SET genes showed increasing expression levels during fruit ripening, and peaked at the turning stage (9 out of 14 genes investigated, Fig. 5B). The expression patterns of those SET genes in fruit development revealed that histone lysine HKMTase genes are dynamically expressed, and that the genomic histone lysine methylation patterns might undergo a dramatic change at the onset of fruit ripening.

### Expression profiles of SET genes during heat/cold shock in *F. vesca*

Histone modifications are suggested to play an important role in the regulation of gene expression in response to abiotic stresses^1^,^14^. Strawberry plants are quite sensitive to extreme temperatures, and cold and heat shock are two recurrent stresses strawberry encounters in the natural environment. To study how HKMTase genes are regulated during heat and cold stresses, the expression patterns of a subset of SET genes were investigated in seedlings. The qRT-PCR results demonstrate that those HKMTase genes respond differentially to a particular abiotic stress (Fig. 5C). Three out of 13 investigated SET genes show increased expression levels upon cold shock at 3 h, while other 10 genes display no significant changes. Two SET genes show increased expressions upon heat shock at 4 h (3 h heat shock + 1 h recovery) as well. Interestingly, *ATX3b* and *SUVH4a* response to both cold and heat shock. Furthermore, the recent duplicated gene pairs response differently. For *SUVH4a/b/c/d*, the expression level of *SUVH4a* increases after cold and heat shock, while the other three are not responsive at all. In summary, some SET genes show dynamic expression patterns upon cold and heat shock, which indicates that these genes may be involved in *F. vesca*’s responses to temperature stresses.

## Discussion

Sequence-based searching and phylogenetic analysis proved to be an effective way to identify histone modifiers in a sequenced genome^20^,^23^,^24^,^25^,^26^,^27^. In this study, we identified genes encoding SET HKMTases, LSD HDMases and JmjC HDMases in *F. vesca* plus seven other plant species representing the major clades of terrestrial plants. The extensive phylogenetic analysis revealed the evolutionary history of those histone modifiers in *F. vesca* and also in other angiosperms.

In total, 45 SET HKMTase genes grouped in seven classes were identified in *F. vesca*. These phylogenetic studies suggest that those identified SET genes were highly conserved in each class across a wide spectrum of plants, indicating their essential regulatory roles in the plant kingdom. Of the SET genes studied, the most intriguing observation was the expansion of class V in both eudicots and monocots, especially in *Z. mays*. Class V SET HKMTases are specific for methylations on H3K9, which is involved in transposon silencing and heterochromatin formation^2^,^5^. There is a vast expansion of transposable elements in *Z. mays*^28^, which might explain the maintenance of a large number of class V SET genes in *Z. mays* to protect the genome integrity. In addition, class V genes diverged faster than other classes (Fig. 3 and Table 2).

For each species, the numbers of genes grouped into each class is summarized in Table 2. Overall, there is no simple linear correlation between SET gene numbers and genome size, or total gene numbers (Table 3). Furthermore, there is no significant difference among the five species in class I, II, III, VI or VII, in terms of gene numbers. It suggests that those five classes did not experience any extensive expansion in angiosperms, and that most of the duplicated genes from the multiple whole genome duplication/triplication events were lost during evolution.

In contrast to the conserved number of SET genes in the five classes mentioned above, class IV and V show distinct evolutionary characteristics. Firstly, class IV SET genes are absent in *A. trichopoda*. Phylogenetic trees suggest that class IV genes are present in *S. moellendorffii* (Supplementary Fig. S7). Thus it is likely that the H3K27me1-specific HKMTase was lost in *A. trichopoda*. Secondly, there is an expansion of class V SET genes in both eudicots and monocots, especially in *Z. mays*. Overall, for the five species investigated, the number of SET genes in class I, II, III, VI and VII remains quite constant, while the number of genes in class IV and V fluctuates in angiosperms, indicating different evolutionary histories accompanied by rounds of WGDs and subsequent gene losses/gains by natural selection constrains.

We identified 26 histone HDMase genes in *F. vesca*. The number of LSD HDMase genes remained nearly the same in eight angiosperm species and the domain construct was highly conserved (Fig. 4B). The JmjC HDMase genes fall into 11 classes indicating that the genes coding for JmjC HDMases underwent rapid expansion and probably functional specification in plant genomes. This expansion and specification process was likely involved in the evolution of epigenetic regulatory mechanisms in plants with distinct biological features. Furthermore, JmjC genes in the KDM3 group were preferentially expanded in the strawberry genome compared to other species, implying that KDM3 group genes may have evolved in strawberry to meet some unique regulatory needs. It is noticeable that there was no H3K79me-specific Dot1/Dot1L HKMTase identified in the five angiosperms (Fig. 1). Considered that *Dot1/Dot1L* gene is present in *S. moellendorffii* (Fig. 1) and also in animals^6^, it is likely that angiosperms have lost the Dot1/Dot1L HKMTases in their common ancestor.

The phylogeny based on both sequence conservation and domain construct in this study suggests that WGDs and dispersed duplications contributed to the expansion of some histone lysine modifiers in angiosperms, which is consistent with that previously reported^20^,^29^. On the other hand, all the *F. vesca* lineage-specific duplications originated from dispersed duplications, particularly retro-transpositions in some cases. The fact that those recently duplicated gene pairs have greatly diverged in expression patterns suggests that they might have been retained in the *F. vesca* genome by selection to more precisely regulate the developmental processes which histone methylation plays a role.

All the SET, LSD and JmjC families have several genes, and their functions could be both redundant and/or complementary. Thus, the overall histone modifications in particular tissues are determined by the combinatorial expression profiles of histone modifiers. Indeed, the different organs/stages have distinct combinatorial expression patterns during flower and fruit development, and many of those histone modifier genes show abrupt up- or down-regulation in specific tissues or developmental stages. Anthers and carpels where sporogenesis and gametogenesis occur, appear to have more genes being up-regulated, irrespective of whether they code for HKMTases or HDMases, highlighting the potentially active and dynamic regulation of histone modifications in these tissues. However, pollen (considered to be in a division- and growth-quiescent state) showed the least number of genes with active expression, particularly the class V SET genes (Fig. 5A). The lack of expression of both H3K9me- and H2K27me2/3-specific SET genes (both for silencing chromatin) is consistent with the de-condensed chromatin states in the vegetative cells in pollen. Pollen grains have three cells, one large vegetative cell and two germ cells. It is known that in *Arabidopsis*, the vegetative cell lack H3K9me2 marks, resulting in the genome-wide activation of transposons. The small RNAs produced by transposon activation are delivered to the germ cells to silence the transposons and thus maintain DNA integrity^30^. The lack of expression of class V SET genes might reflect this compromised strategy of the large vegetative cell to protect the germ lines. A few genes coding for demethylase were sharply up-regulated in specific stages or tissues, for example *FV*_*JmjC9* and *FV*_*JmjC22* in pollen, which might serve critical regulatory function there.

Another interesting phenomenon is that the expression levels of the some active SET genes that we investigated in pith and cortical tissues decreased during early-stage fruit development (from pollination to big green stage), but gradually increased beginning at the white stage and reaching a peak at the turning stage (Fig. 5). It has been reported that epigenetic marks fluctuate during fruit development and ripening, *e.g*. overall DNA methylation levels decrease during tomato fruit ripening^31^. Global DNA methylation measurements revealed that DNA methylation varied in both peal and flesh of sweet orange^32^. Our results suggest that histone modifications might be dynamic during strawberry fruit development and ripening as well, and histone modifiers are probably involved in this regulatory process.

Epigenentic regulation of cellular processes during abiotic stresses has been the subject of several recent investigations that suggest that both DNA modifications and histone modifications play a pivotal role in plant responses to various stresses^1^,^14^,^33^,^34^. The RT-qPCR results revealed that the expression levels of a set of HKMTases were found to be elevated after cold/heat shock (Fig. 5C), indicating their possible participation in response to extreme temperatures in strawberry plants. Overall, expressions of histone modifers are dynamic in different tissues during different developmental stages, and in response to abiotic stresses as well, demonstrating their regulatory roles in various cellular processes in strawberry.

Compared to the well-studied model plants *Arabidopsis* and rice, strawberry has several distinct characteristics including that “fruits” develop from receptacle tissues and the adaptability of the species to different environments. As essential regulatory factors, how histone modifiers are involved in various cellular process is of great interest. The majority of previous studies about histone modifiers focused on *Arabidopsis*, which does not have fleshy fruits. Although some work has been published about histone modifiers in tomato, the data are limited. Our identification and characterization of histone modifiers in woodland strawberry is the first comprehensive analysis of HKMTases and HDMases in a non-climacteric fruit species. Our study provides an overview of the histone lysine methylation modifiers in strawberry, and should greatly facilitates molecular, biochemical and physiological characterizations of histone methylations in strawberry and other *Rosaceae* species.

## Methods

### Data retrieve

Eight plant genomes were analyzed, *Fragiaria vesca*, *Arabidopsis thaliana*, *Vites vinifera, Nelumbo nucifera*, *Orazy sativa*, *Zea mays*, *Amborella trichopoda* and *Selaginella moellendorffii*. The *Fragaria vesca* and *Nelumbo nucifera* complete protein sequences and corresponding annotation information were downloaded from NCBI and others were download from Phytozome (version 10.3; http://phytozome.jgi.doe.gov/pz/portal.html). See Supplementary Table S5 for versions and resources of the databases. In proteome datasets, if more than one protein are annotated for the same gene from alternative splicing, the longest form was used for further analysis.

### Identification of genes with the domain(s) of interest

To identify the genes with the domain(s) of interest, the amino acid sequences of SET domain (PF00856), JmjC domain (PF02373), Amino_oxidase domain (PF05193) and SWIRM domain (PF04433) were downloaded from pfam database V27.0^35^ and used as a query to find homologous sequences in proteome datasets, respectively. To verify the presence of those domains, the resulting sequences were verified using the Pfam database (http://pfam.xfam.org/search), Conserved Domain Database^36^ (CDD; http://www.ncbi.nlm.nih.gov/Structure/cdd/wrpsb.cgi) available from NCBI, and the Simple Modular Architecture Research Tool database^37^ (SMART; http://smart.embl-heidelberg.de/), with a threshold of e-value < 1e−4. Proteins with both Amino_oxidase domain and SWIRM domain were identified as LSD genes.

### Sequences alignment and phylogenetic analysis

Protein sequences were aligned using MUSCLE v3.8.31 with default parameters^38^. Phylogenetic trees were constructed by Raxml (version 8.1.16) with gamma distribution and 1000 bootstrapping replicates^39^. The construct of each phylogenetic tree was verified by MrBayes v3.2.4^40^.

### Domain and motif analysis, and identification of *F. vesca* lineage-specific duplicated pairs

All identified proteins were used to search against the PFAM, SMART and CDD databases to search for other known domains. All domains found by any of the three databases with e-value < 10^−4^ were kept. In addition, motif analyses were performed online by MEME (MEME, Version4.10.2, http://meme-suite.org/tools/meme)^41^. The number of motifs was set at no more than 20 with the length from 15–50 amino acids for each search. The lineage-specific duplicated gene pairs were identified based on both phylogenetic trees, domain composition of the whole proteins and motif composition of the SET domains. *FV-SET13/35* was not identified as an *F. vesca* lineage-specific duplicated pair by the phylogenetic tree constructed for SET class V (Supplementary Fig. S4) and thus was omitted; while *FV-SET33/40* and *FV-SET10/12* were included based on either domain composition (Supplementary Fig. S2) or phylogenetic trees constructed for each SET class (Supplementary Fig. S6).

### Plant growth conditions, stress treatments and material collection

A 7th generation inbred line of woodland *Fragaria vesca*, Ruegen F7-4 (Kindly provided by Janet Slovin) was used for all strawberry material collection. Strawberry flesh at different development stages was collected from plants grown in 10 cm x 10 cm pots in a controlled-environment growth chamber, set at 16 h light/8 h dark cycles, 22 °C, and 65% relative humidity. Strawberry fruits at 12-day old big green stage, big white stage (white flesh with red seeds), pink stage (slight pink flesh and red seeds), and red stage (2–3 days after the pink stage) were collected and immediately put into liquid nitrogen.

The strawberry seedlings for cold/heat shock were grown in MS media in a growth chamber set at 16 h light/8 h dark cycles, 22 °C. Four-week old seedlings were transferred to a growth chamber set at either 4 °C or 38 °C for cold/heat shock. Cold shocked seedlings were collected at 1 h, 3 h and 8 h; while heat shocked seedlings were collected at 1 h and 3 h, or at 4 h (3 h heat shock and 1 h recovery at 22 °C) and 8 h (3 h heat shock and 5 h recovery at 22 °C). The collected materials were immediately put into liquid nitrogen for RNA processing.

### RNA extraction and expression analysis

Before RNA extraction, the achenes were stripped from the frozen strawberry fruit, and only the de-seeded flesh was processed for RNA isolation. RNA was isolated from either the deseeded flesh or seedlings by a modified CTAB method. After DNase I treatment, RNAs were used for cDNA synthesis by using the Primerscript RT reagent Kit with gDNA Erase (Takara). The cDNAs were used as templates for quantitative RT-PCR to measure the abundance of a certain transcript. Quantitative RT-PCR was performed using SYBR Premix Ex Tag (Takara) on a Bio-rad iQ5. Primers used are listed in Supplementary Table S6. Results were analyzed by using the ΔΔCT method^42^ using GAPDH as the control locus^43^. Three biological and three technical replicates were performed and analyzed.

## Additional Information

**How to cite this article**: Gu, T. *et al*. Identification and characterization of histone lysine methylation modifiers in *Fragaria vesca*. *Sci. Rep*. **6**, 23581; doi: 10.1038/srep23581 (2016).

## Supplementary Material

Supplementary Information

## Figures and Tables

**Table 1:** Characterization of SET-domain containing genes in *F. vesca*.

Class	Name	Annotation	Locus tag	Specificity	Name	Annotation	Locus tag	ProteinLength(aa)	Chr
	A*. thaliana*				*F. vesca*				
I	AT-MEDEA	MEA	AT1G02580.1	H3K27me3	–	–	–	–	–
	AT-EZA1	SWN	AT4G02020.1	H3K27me3	FV-SET29	EZA	gene02275	880	5
	AT-CLF	CLF	AT2G23380.1	H3K27me3	FV-SET4	CLF	gene23813	913	1
II	AT-ASHH1	ASHH1	AT1G76710.1	H3;H4	FV-SET38	ASHH1	gene00440	1522	7
	AT-ASHH2	ASHH2	AT1G77300.1	H3K4me3	FV-SET15	ASHH2	gene11269	2113	2
	AT-ASHH3	ASHH3	AT2G44150.1	–	FV-SET1FV-SET6	ASHH3–	gene30492#gene30492#	372372	11
	AT-ASHH4	ASHH4	AT3G59960.1	–	–	–	–	–	–
	AT-ASHR3	ASHR3	AT4G30860.1	H3K4me2H3K36me2/me3	FV-SET25	ASHR3	gene27657	485	4
III	AT-SDG25	ATXR7	AT5G42400.1	H3K4me1/me2/me3	FV-SET32	–	gene29324	1229	5
	AT-ATXR3	ATXR3	AT4G15180.1	H3K4me1/me2/me3	FV-SET2	ATXR3	gene23883	2402	1
	AT-ATX1AT-ATX2	ATX1ATX2	AT2G31650.1AT1G05830.1	H3K4me3H3K4me2	FV-SET24	ATX2	gene22427	1075	4
	AT-ATX3	ATX3	AT3G61740.1	–	FV-SET8FV-SET39	ATX3aATX3b	gene22028gene19196	756908	17
	AT-ATX4AT-ATX5	ATX4ATX5	AT4G27910.1AT5G53430.1	–	FV-SET42	ATX5	gene12861	1069	7
	–	–	–	–	FV-SET16	–	gene10999	2170	2
IV	AT-ATXR5	ATXR5	AT5G09790.2	H3K27me1	FV-SET7	ATXR5	gene17873	379	1
	AT-ATXR6	ATXR6	AT5G24330.1	H3K27me1	FV-SET41	ATXR6	gene12820	344	7
V	AT-SUVH1AT-SUVH3AT-SUVH7AT-SUVH8AT-SUVH8	SUVH1SUVH3SUVH7SUVH8SUVH10	AT5G04940.1AT1G73100.1AT1G17770.1AT2G24740.1AT2G05900.1	–	FV-SET33FV-SET40	SUVH1aSUVH1b	gene02482gene03234	702664	67
	AT-SUVH2AT-SUVH9	SUVH2SUVH9	AT2G33290.1AT4G13460.1	H3K9me1/me2H4K20meH3K27me2–	FV-SET14	SUVH9	gene08379	674	2
	AT-SUVH4	SUVH4	AT5G13960.1	H3K9me1/me2	FV-SET34	SUVH4a	gene01396	651	6
					FV-SET26FV-SET45FV-SET18	SUVH4bSUVH4cSUVH4d	gene06630gene07293gene19999	406639412	4unknown3
	AT-SUVH5AT-SUVH6	SUVH5SUVH6	AT2G35160.1AT2G22740.2	H3K9me1/me2H3K9me1/me2	FV-SET30	SUVH6	gene20484	1083	5
	AT-SUVR1AT-SUVR2	SUVR1SUVR2	AT1G04050.1AT5G43990.2	–	FV-SET13	SUVR2	gene08324	825	2
	AT-SUVR3	SUVR3	AT3G03750.2	–	FV-SET11FV-SET17	SUVR3aSUVR3b	gene11265gene21746	340346	22
	AT-SUVR4	SUVR4	AT3G04380.1	H3K9me2/me3	FV-SET35	SUVR4a	gene16684	511	6
	–	–	–	–	FV-SET44FV-SET22	SUVR4u1SUVR4u2	gene07805gene11562	8121303	unknown4
	AT-SUVR5	SUVR5	AT2G23740.2	H3K9me2H3K27me2	FV-SET31	SUVR5	gene29411	1520	5
VI	AT-ASHR2	ASHR2	AT2G19640.2	–	FV-SET20	ASHR2	gene22755	395	4
	AT-ATXR2	ATXR2	AT3G21820.1	–	FV-SET3	ATXR2	gene23908	472	1
	AT-RE-PR	–	AT1G33400.1	–	–	–	–	–	–
	AT-SET38	–	AT5G06620.1	–	FV-SET36	ATXR4	gene18071	327	6
	AT-SETC4	–	AT1G43245.1	–	FV-SET9	-	gene08123	646	2
	AT-SET35	–	AT1G26760.1	–	FV-SET28	-	gene04715	522	4
	AT-SET37	–	AT2G17900.1	–	FV-SET10FV-SET12	ASHR1aASHR1b	gene11111gene25503	483525	22
VII	AT-SET40	–	AT5G17240.1	–	FV-SET5	–	gene31667	512	1
	AT-RUB-ME	–	AT3G07670.1	–	FV-SET37	–	gene15541	499	6
	AThal9	–	AT1G14030.1	–	FV-SET27	–	gene04693	598	4
	AT-PLAS	–	AT4G20130.1	–	FV-SET19	–	gene19947	514	3
	AT-LRU-PR	–	AT1G24610.1	–	FV-SET21	–	gene06267	483	4
	AT-SETC1	–	AT1G01920.1	–	FV-SET23	–	gene16946	563	4
	AT-SETC2	–	AT3G55080.1	–	FV-SET43	–	gene23354	461	7
	AT-SETC3	–	AT3G56570.1	–	–	–	–	–	–

^#^It is noted that FV-SET1 and FV-SET6 have the same gene tag (gene30492) by “Hybrid GeneMArk Predictions” (predicted by EST data, https://www.rosaceae.org/species/fragaria/fragaria_ vesca/genome_ v1.0). But the genomic assembly indicates that these two genes are located at different loci on chromosome 1 (Fig. 3A) and different XP tags (Supplementary Table S1). In addition, these two genes have different intron/exon structures and DNA sequences (Supplementary Fig. S1). Thus, we retained both of these genes.

**Table 2:** Characterization of JmjC and LSD histone HDMase encoding genes in *F. vesca*.

Class	Name	Annotation*	Locus tag	Name	Locus tag	ProteinLength(aa)	Chr
	*A. thaliana*			*F.vesca*			
PKDM7	AT-JmjC9	PKDM7A	AT2G38950	FV_JmjC2	gene08108	859	2
	AT-JmjC15AT-JmjC8AT-JmjC4	PKDM7BPKDM7CPKDM7E	AT4G20400AT2G34880.1AT1G30810.1	FV_JmjC5	gene20210	1069	3
	AT-JmjC1	PKDM7D	AT1G08620	FV_JmjC15	gene16665	1218	6
PKDM9	AT-JmjC13	PKDM9A	AT3G48430.1	FV_JmjC10	gene09903	1492	5
	AT-JmjC17	PKDM9B	AT5G04240.1	FV_JmjC18	gene23255	1590	7
PKDM11	AT-JmjC21	PKDM11	AT5G63080.1	FV_JmjC8	gene31779	475	5
PKDM12	AT-JmjC11	PKDM12A	AT3G20810.2	FV_JmjC6	gene20651	416	3
	AT-JmjC19	PKDM12B	AT5G19840.2	FV_JmjC1	gene20265	563	2
PKDM13	AT-JmjC12	PKDM13	AT3G45880.1	FV_JmjC3	gene10362	348	3
KDM3	AT-JmjC2	KDM3A	AT1G09060.3	FV_JmjC16	gene22503	965	6
	AT-JmjC16	KDM3B	AT4G21430.1	–	–	–	–
	AT-JmjC10	KDM3C	AT3G07610.3	FV_JmjC14FV_JmjC13FV_JmjC20FV_JmjC12FV_JmjC21	gene22017gene27692gene04808gene11798gene09210	4459601234876804	55757
	AT-JmjC14	KDM3D	AT4G00990.1	FV_JmjC22	gene12874	882	7
	–	–	–	FV_JmjC19	gene19156	1017	7
	AT-JmjC3AT-JmjC5	KDM3EKDM3F	AT1G11950.1AT1G62310.1	FV_JmjC4	gene19492	867	3
KDM5	AT-JmjC6	KDM5	AT1G63490.1	FV_JmjC7	gene32474	1839	5
PKDM8	AT-JmjC20	KDM8	AT5G46910.1	FV_JmjC9	gene13745	878	5
PKDM6	AT-JmjC18	JMJD6A	AT5G06550.1	FV_JmjC17	gene18131	519	6
	AT-JmjC7	JMJD6B	AT1G78280.1	FV_JmjC11	gene11964	959	5
LSD	AT-LSD1	–	AT1G62830.1	FV-LSD1	gene08618	791	2
	AT-LSD2	–	AT3G10390.1	FV-LSD2	gene23463	911	7
	AT-LSD3	–	AT3G13682.1	FV-LSD3	gene25010	748	3
	AT-LSD4	–	AT4G16310.1	FV-LSD4	gene15221	1863	2

^*^Refer to Qian *et al*.^20^ for annotations of JmjC demethylase genes.

**Table 3:** Number of SET genes identified in each class in the five species.

	SET	I	II	III	IV	V	VI	VII	Genes	Genome	WGD*
*A.trichopoda*	34	3	4	5	0	10	5	7	26846	701M	2
*O. sativa*	44	2	5	6	2	14	7	8	39049	373M	5
*Z. mays*	54	4	6	6	2	21	8	7	63480	2053M	6
*A. thaliana*	47	3	5	7	2	15	7	8	27416	118M	5
*F. vesca*	45	2	5	7	2	15	7	7	24771	212M	3

^*^Refer to Fig. 1 for more details of WGDs.
