# Supplementary material for: Identification and characterization of histone lysine methylation modifiers in Fragaria vesca
Source: Sci Rep. 2016 Apr 6;6:23581. doi: 10.1038/srep23581 (PMC4822149; doi:10.1038/srep23581)

Identification and characterization of histone lysine methylation modifiers in *Fragaria vesca*

Tingting Gu, Yuhui Han, Ruirui Huang, Richard J. McAvoy, Yi Li

Supplementary Table S1 Locus tags for SET genes

Class	Name	Locus tag(GDR)	Locus tag(NCBI)
**I**	FV-SET29	gene02275	XP_004300446.1
	FV-SET4	gene23813	XP_004288020.1
**II**	FV-SET38	gene00440	XP_004306471.1
	FV-SET15	gene11269	XP_004292727.1
	FV-SET1	gene30492	XP_004287196.1
	FV-SET6	gene30492	XP_004288600.1
	FV-SET25	gene27657	XP_004298060.1
**III**	FV-SET32	gene29324	XP_004301597.1
	FV-SET2	gene23883	XP_004287912.1
	FV-SET24	gene22427	XP_004298031.1
	FV-SET16	gene10999	XP_004292737.1
	FV-SET8	gene22028	XP_004289558.1
	FV-SET39	gene19196	XP_004306675.1
	FV-SET42	gene12861	XP_004307977.1
**IV**	FV-SET7	gene17873	XP_004289409.1
	FV-SET41	gene12820	XP_004307957.1
**V**	FV-SET33	gene02482	XP_004304345.1
	FV-SET40	gene03234	XP_004307309.1
	FV-SET34	gene01396	XP_004304567.1
	FV-SET26	gene06630	XP_004298233.1
	FV-SET45	gene07293	XP_004310138.1
	FV-SET18	gene19999	XP_004295500.1
	FV-SET30	gene20484	XP_004300536.1
	FV-SET14	gene08379	XP_004292255.1
	FV-SET13	gene08324	XP_004292239.1
	FV-SET11	gene11265	XP_004290913.1
	FV-SET17	gene21746	XP_004293056.1
	FV-SET35	gene16684	XP_004304779.1
	FV-SET44	gene07805	XP_004309929.1
	FV-SET22	gene11562	XP_004297848.1
	FV-SET31	gene29411	XP_004300581.1
**VI**	FV-SET20	gene22755	XP_004296999.1
	FV-SET3	gene23908	XP_004287921.1
	FV-SET36	gene18071	XP_004305542.1
	FV-SET9	gene08123	XP_004290505.1
	FV-SET28	gene04715	XP_004298417.1
	FV-SET10	gene11111	XP_004290832.1
	FV-SET12	gene25503	XP_004292117.1
**VII**	FV-SET5	gene31667	XP_004288574.1
	FV-SET37	gene15541	XP_004305682.1
	FV-SET23	gene16946	XP_004297873.1
	FV-SET27	gene04693	XP_004298409.1
	FV-SET19	gene19947	XP_004295521.1
	FV-SET21	gene06267	XP_004297442.1
	FV-SET43	gene23354	XP_004308826.1

Supplementary Table S2. Synteny and collinearity analysis for SET, JmjC and LSD genes. The possible mechanisms (WGD, tandem duplication or others) responsible for the duplication events were analyzed by MCscan with default settings (Wang et al., 2012)

	SET genes					
	Singleton	Dispersed	Proximal	Tandem	WGD/Segmental	Total
Amborella trichopoda	5	27	2	0	0	34
Oryza sativa	7	30	0	2	5	44
Zea mays	19	29	0	0	6	54
Arabidopsis thaliana	14	20	1	0	12	47
Fragaria vesca	4	35	0	0	6	45

	JmjC genes					
	Singleton	Dispersed	Proximal	Tandem	WGD/Segmental	Total
Amborella trichopoda	3	13	0	1	0	17
Oryza sativa	2	13	0	0	2	17
Zea mays	17	9	0	0	0	26
Arabidopsis thaliana	1	15	0	0	5	21
Fragaria vesca	0	20	0	0	2	22

	LSD genes					
	Singleton	Dispersed	Proximal	Tandem	WGD/Segmental	Total
Amborella trichopoda	0	5	0	0	0	5
Oryza sativa	0	4	0	0	0	4
Zea mays	0	4	0	0	0	4
Arabidopsis thaliana	0	4	0	0	0	4
Fragaria vesca	0	4	0	0	0	4

Supplementary Table S3. Duplicated SET and JmjC gene pairs identified by Mcscan as resulted from whole genome duplication (WGD) in *F. vesca.*

Species	Collinearity (Gene ID)	
	FV-SET39(ATX3b) (gene19196)	FV-SET42(ATX5) (gene12861)
Fragaria vesca	FV-SET13(SUVR2) (gene08324)	FV-SET35(SUVR4a) (gene16684)
	FV-SET13(SUVR2) (gene08324)	FV-SET22(SUVR4u2) (gene11562)
	FV-SET34 (gene01396)	Other gene (gene06398)[#]
	FV-SET34 (gene01396)	Other gene (gene07306)[#]
	Fv_JmjC19 (gene19156)	FV_JmjC22 (gene12874)

[#] Genes identified by MCscan as WGD-related duplicated genes, but have lost the SET or JmjC domain already. Thus, those genes were not found by our sequence-based searching as SET domain- or JmjC domain-containing genes.

Supplementary Table S4 Locus tags for JmjC and LSD genes

Class	Name	Locus tag(GDR)	Locus tag(NCBI)
**PKDM7**	FV-JmjC2	gene08108	XP_004292214.1
	FV-JmjC5	gene20120	XP_004295454.1
	FV-JmjC15	gene16665	XP_004302095.1
**PKDM9**	FV-JmjC10	gene09903	XP_004301036.1
	FV-JmjC18	gene23255	XP_004307375.1
**PKDM11**	FV-JmjC8	gene31779	XP_004298971.1
**PKDM12**	FV-JmjC6	gene20651	XP_004296345.1
	FV-JmjC1	gene20265	XP_004290635.1
**PKDM13**	FV-JmjC3	gene10362	XP_004294223.1
**KDM3**	FV-JmjC16	gene22503	XP_004302409.1
	FV-JmjC14	gene22017	XP_004301847.1
	FV-JmjC13	gene27692	XP_004301767.1
	FV-JmjC20	gene04808	XP_004308523.1
	FV-JmjC12	gene11798	XP_004301534.1
	FV-JmjC21	gene09210	XP_004308619.1
	FV-JmjC22	gene12874	XP_004309136.1
	FV-JmjC19	gene19156	XP_004308306.1
	FV-JmjC4	gene19492	XP_004295292.1
**KDM5**	FV-JmjC7	gene32474	XP_004298791.1
	FV-JmjC9	gene13745	XP_004300206.1
**JMJD6**	FV-JmjC17	gene18131	XP_004303402.1
	FV-JmjC11	gene11964	XP_004301326.1
**LSD**	FV-LSD1	gene08618	XP_004292921.1
	FV-LSD2	gene23463	XP_004308766.1
	FV-LSD3	gene25010	XP_004294498.1
	FV-LSD4	gene15221	XP_004293193.1

Supplementary Table S5 Databases used in this study.

Species	Version	Resources
Amborella trichopoda	Version 1.0	Phytozome V10.3
Arabidopsis thaliana	TAIR 10	Phytozome V10.3
Oryza sativa	Version 7.0	Phytozome V10.3
Zea mays	Version 6a	Phytozome V10.3
**Vites vinifera**	Version 12X	Phytozome V10.3
**Selaginella moellendorffii**	Version 1.0	Phytozome V10.3
Fragaria vesca	Version 1.0	http://www.ncbi.nlm.nih.gov/assembly/GCF_000184155.1/
**Nelumbo nucifera**	Version 1.1	http://www.ncbi.nlm.nih.gov/assembly/GCF_000365185.1

Supplementary Table S6. Primers used for quantitative studies.

Primer	Sequences
qpcr-Fvesc-EZA-Forward	TGGATATGGTTGGACAGAAATCA
qpcr-Fvesc-EZA-Reverse	CTAGAGCTTCAACGCCATGT
qpcr-Fvesc-CLF-forward	GGTGATGAAAGTAATGGGAGCA
qpcr-Fvesc-CLF-reverse	CAGCATTCTTGACAGCAACATTAG
qpcr-Fvesc-atx3b-forward	ATTGAGGAGGCAGTGTTAGC
qpcr-Fvesc-atx3b-reverse	TCTGTGGCCTCTTGGATTTC
qpcr-Fvesc-atx5-forward	GGAAGAAGAGGAAGAAGGGTATATG
qpcr-Fvesc-atx5-reverse	CCGTCTTTCCTTGAAGTGAGT
qpcr-Fvesc-atxr5-forward	TCCGCCGAAGAAGTTCAAG
qpcr-Fvesc-atxr5-reverse	GCACCTGACGTCACTGTAAT
qpcr-Fvesc-atxr6-forward	GATTCAGAGAAACGTCGAAACAC
qpcr-Fvesc-atxr6-reverse	ACACCACCAAACTACTTCCC
qpcr-Fvesc-suvh1a-forward	CATTTAGTGCACCACCACAAG
qpcr-Fvesc-suvh1a-reverse	CCAGCTGGTGTTTGCATTG
qpcr-Fvesc-suvh1b-forward	CAGGAGGTCAAGACTCCATTC
qpcr-Fvesc-suvh1b-reverse	AGGAACAAGACGCCTCAAA
qpcr-Fvesc-suvh4a-forward	ACAAGAGTGACCGTGTTAGG
qpcr-Fvesc-suvh4a-reverse	CCTCTTCTCTTCTTCCTGTACG
qpcr-Fvesc-suvh4c-forward	GGATAAGGATTGTGAGGGTGAG
qpcr-Fvesc-suvh4c-reverse	TTGCCTTGGTCATCGATTCT
qpcr-FV26-SUVH4b-F	GAGCAACAGCTCTATTACCTCTATC
qpcr-FV26-SUVH4b-R	ATTCTCATCAACCTTGGAAGAAGAC
qpcr-Fvesc-suvr2-forward	GAGGCAGAGGAACTACTTGATG
qpcr-Fvesc-suvr2-reverse	GAGGTTGCTTGCCCTTATCT
qpcr-Fvesc-ashr1a-forward	AAATCGTTCTCCGCGATTCT
qpcr-Fvesc-ashr1a-reverse	AAGCAGTGGTCGCAGTAAG
qpcr-Fvesc-ashr2-forward	TGAAGGCAATGGAGACACTC
qpcr-Fvesc-ashr2-reverse	ATTCTTTATAAGCTCCTCCCAAGT
qpcr-GAPDH2-F	CATTCATCACCACCGACTACA
qpcr-GAPDH2-R	GAAGGGTCTTCTCATCCTTGAC

Supplementary Figure S1. A most likelihood phylogenetic tree, domain compositions and exon/intron constructions of the predicted SET genes identified in *F. vesca* and *A. thaliana*. The phylogenetic tree was constructed based on the amino acids sequences of the SET domains with 1000 bootstrapping replicates.

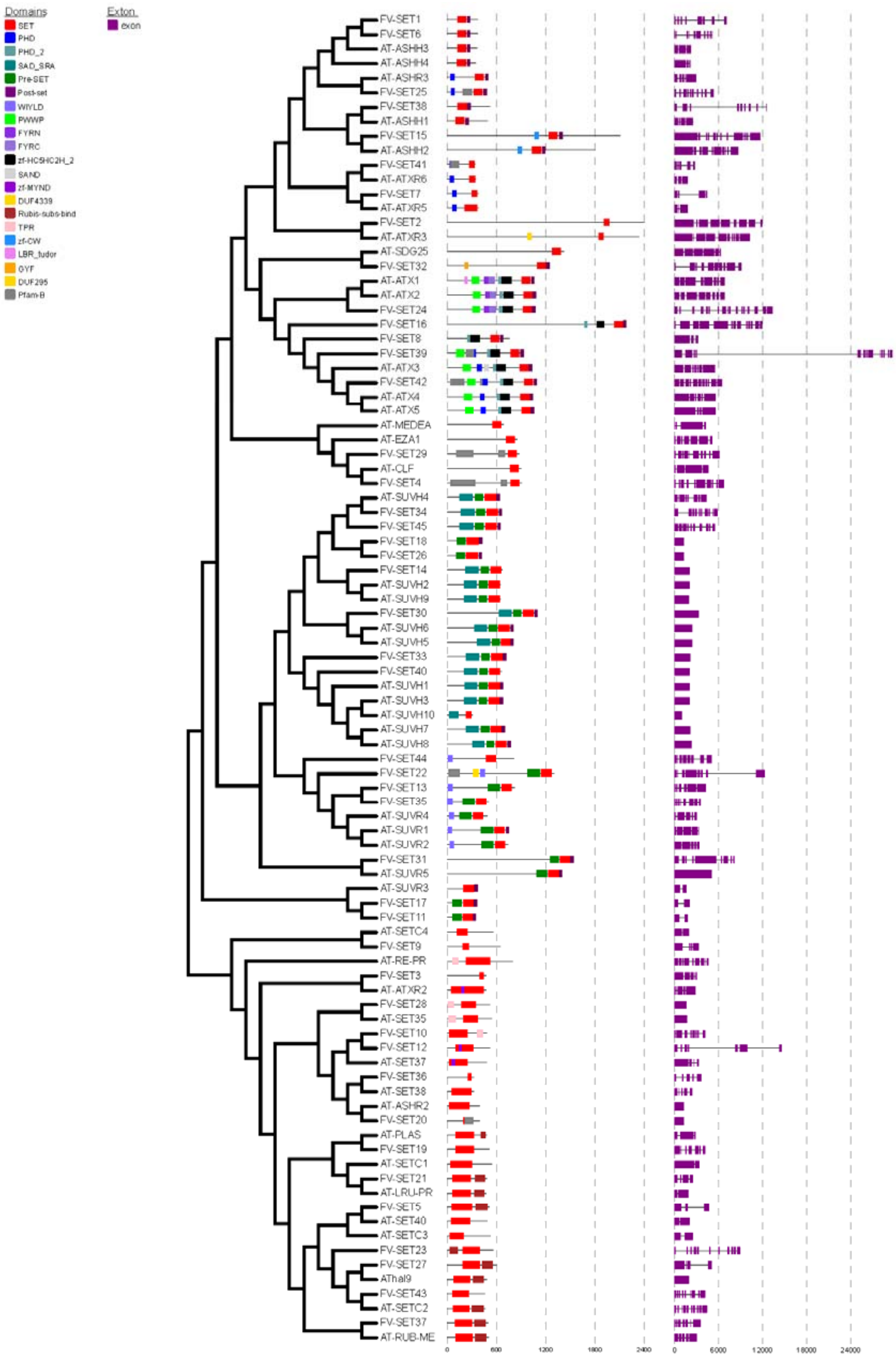

Supplementary Figure S2. Motif composition of SET domains of the class I-V SET genes identified in *F. vesca* and *A. thaliana.* Motif analysis was performed online by MEME (Version4.10.2). The number of motifs was set no more than 20 with the length from 15-50 amino acids.

Subfamily	Motif	1	2	3	4	5	6	7	8	9	10	11	12	13	14	15	16	17	18	19	20
I																					
	AT-MEDEA	1	1	1	1	0	0	0	0	0	0	0	0	0	0	0	1	0	0	0	0
	AT-EZA1	0	0	1	1	0	0	0	0	0	0	0	0	0	0	0	1	1	0	0	0
	AT-CLF	0	0	1	1	0	0	0	0	1	0	0	0	0	1	0	1	1	0	0	0
	FV-SET29	0	0	1	1	0	0	1	0	0	0	0	0	0	0	0	1	1	1	0	0
	FV-SET4	0	0	0	1	0	0	0	0	0	0	0	1	0	0	0	1	1	0	0	0
II																					
	AT-ASHH1	1	1	1	1	0	0	0	0	1	0	0	0	0	0	0	0	0	0	0	0
	AT-ASHH2	1	1	1	1	0	0	0	0	1	0	0	0	0	0	0	0	0	0	0	1
	AT-ASHH3	1	1	1	1	0	0	0	0	1	0	0	0	0	0	0	0	0	0	0	1
	AT-ASHH4	1	1	1	1	0	0	0	0	1	0	0	0	0	0	0	0	0	0	0	1
	AT-ASHR3	1	1	1	1	0	0	0	0	1	0	0	0	0	0	0	0	0	0	0	1
	FV-SET38	1	1	1	1	0	0	0	0	1	0	0	0	0	0	0	0	0	1	0	0
	FV-SET15	1	1	1	1	1	0	0	0	1	0	1	0	0	0	0	1	0	0	0	1
	FV-SET6	1	1	1	1	0	0	0	0	1	0	0	0	0	0	0	0	0	0	0	1
	FV-SET1	1	1	1	1	0	0	0	0	1	0	0	0	0	0	0	0	0	0	0	1
	FV-SET25	1	1	1	1	0	0	0	0	1	0	0	0	0	0	0	0	0	0	0	1
III																					
	AT-ATX2	1	1	1	0	0	0	1	1	1	0	0	0	0	0	1	0	0	0	0	0
	AT-ATX1	1	1	1	0	0	0	1	1	1	0	0	0	0	0	1	0	0	0	0	0
	AT-ATX3	1	1	1	0	0	0	1	1	1	0	0	1	0	0	1	0	0	0	1	0
	AT-ATX4	1	1	1	0	0	0	1	1	1	0	0	1	0	0	1	1	0	0	1	0
	AT-ATX5	1	1	1	0	0	0	1	1	1	0	0	1	0	0	1	0	0	0	1	0
	AT-ATXR3	1	0	1	0	0	0	0	0	1	0	0	0	1	1	0	0	0	0	0	0
	FV-SET32	1	1	1	1	0	0	0	0	1	1	0	0	0	0	0	0	0	0	1	0
	FV-SET16	1	1	1	1	0	0	1	1	1	0	0	0	0	0	0	0	0	0	0	0
	FV-SET2	1	1	1	1	0	0	0	0	1	1	0	0	0	0	0	0	0	1	0	0
	FV-SET39	1	1	1	0	0	0	1	1	1	0	0	1	0	0	1	0	0	0	1	0
	FV-SET8	1	1	1	0	0	0	1	1	1	0	0	1	0	0	0	0	0	0	1	0
	FV-SET24	1	1	1	0	1	0	1	1	1	0	0	0	0	1	0	0	0	0	0	0
	FV-SET42	1	1	1	1	0	0	1	1	1	0	0	1	0	0	1	0	0	0	1	0
IV																					
	AT-ATXR6	0	1	0	0	0	0	0	0	0	0	0	0	0	0	0	1	0	1	0	0
	AT-ATXR5	1	1	0	0	0	0	0	0	0	0	0	0	0	0	0	0	0	1	0	0
	FV-SET7	1	1	0	0	0	0	0	0	0	0	0	0	0	0	0	0	0	1	0	0
	FV-SET41	1	1	0	0	0	0	0	0	0	0	0	0	0	0	0	0	0	1	0	0
V																					
	AT-SUVH1	1	1	1	1	1	1	0	0	1	1	1	0	1	1	0	0	0	0	0	0
	AT-SUVH2	1	1	1	1	1	1	0	0	0	1	1	0	1	1	0	0	0	0	0	0
	AT-SUVH3	1	1	1	1	1	1	0	0	1	1	1	0	1	1	0	0	0	0	0	0
	AT-SUVH4	1	1	1	1	1	1	0	0	1	1	1	0	1	1	0	0	0	0	0	0
	AT-SUVH5	1	1	1	1	1	1	0	0	1	1	1	0	1	1	0	0	0	0	0	0
	AT-SUVH6	1	1	1	1	1	1	0	0	1	1	1	0	1	1	0	0	0	0	0	0
	AT-SUVH7	1	1	1	1	1	1	0	0	1	1	1	0	1	1	0	0	0	0	0	0
	AT-SUVH8	1	1	1	0	1	1	0	0	1	1	1	0	1	1	0	0	0	0	0	0
	AT-SUVH9	1	1	1	1	1	1	0	0	0	1	1	0	1	1	0	0	0	0	0	0
	AT-SUVH10	1	0	1	0	1	0	0	0	1	1	1	0	1	0	0	0	0	0	0	0
	AT-SUVR1	1	1	1	1	0	0	0	0	1	0	0	0	1	1	0	0	0	0	0	0
	AT-SUVR2	1	1	1	1	0	0	0	0	1	0	0	0	1	0	0	1	0	0	0	0
	AT-SUVR3	1	1	1	1	0	0	0	0	1	0	0	0	1	0	0	0	0	0	0	0
	AT-SUVR4	1	1	1	1	0	0	0	0	1	0	0	0	1	1	0	0	0	0	0	0
	AT-SUVR5	1	1	1	1	0	0	0	0	1	0	0	0	1	0	0	0	0	0	0	0
	FV-SET33	1	1	1	1	1	1	0	0	1	1	1	0	1	1	0	0	0	0	0	0
	FV-SET40	1	1	1	1	1	1	0	0	1	1	1	0	1	1	0	0	0	0	0	0
	FV-SET34	1	1	1	1	1	1	0	0	1	1	1	0	1	1	0	0	0	0	0	0
	FV-SET45	1	1	1	1	1	1	0	0	1	1	1	0	1	1	0	0	0	0	0	0
	FV-SET26	1	1	1	1	0	0	0	0	1	0	0	0	1	1	0	0	0	0	0	0
	FV-SET18	1	1	1	1	0	0	0	0	1	0	0	0	1	1	0	0	0	0	0	0
	FV-SET30	1	1	1	1	1	1	0	0	1	1	1	0	1	1	0	0	0	0	0	0
	FV-SET14	1	1	1	1	1	1	0	0	0	1	1	0	1	1	0	0	0	0	0	0
	FV-SET13	1	1	1	1	0	0	0	0	1	1	0	0	1	1	0	0	0	0	0	0
	FV-SET11	1	1	1	1	0	0	0	0	1	0	0	0	1	0	0	0	0	0	0	0
	FV-SET17	1	1	1	1	0	0	0	0	1	0	0	0	1	0	0	0	0	0	0	0
	FV-SET35	1	1	1	1	0	0	0	0	1	0	0	0	1	1	0	0	0	0	0	0
	FV-SET44	1	1	1	1	0	0	0	0	1	0	0	0	0	1	0	0	0	0	0	0
	FV-SET22	1	1	1	1	0	0	0	0	1	1	0	0	1	1	0	0	0	0	0	0
	FV-SET31	1	1	1	1	0	0	0	1	1	0	0	0	1	0	0	0	0	1	0	0

Supplementary Figure S3. A most likelihood phylogenetic tree and domain composition of the class I-IV SET genes identified in the five species (*F. vesca*, *A. thaliana*, *O. sativa*, *Z. mays* and *A. trichopoda*). The phylogenetic tree was constructed based on the amino acids sequences of the whole proteins with 1000 bootstrapping replicates, and the results of the bootstrapping analysis larger than 50% are shown.

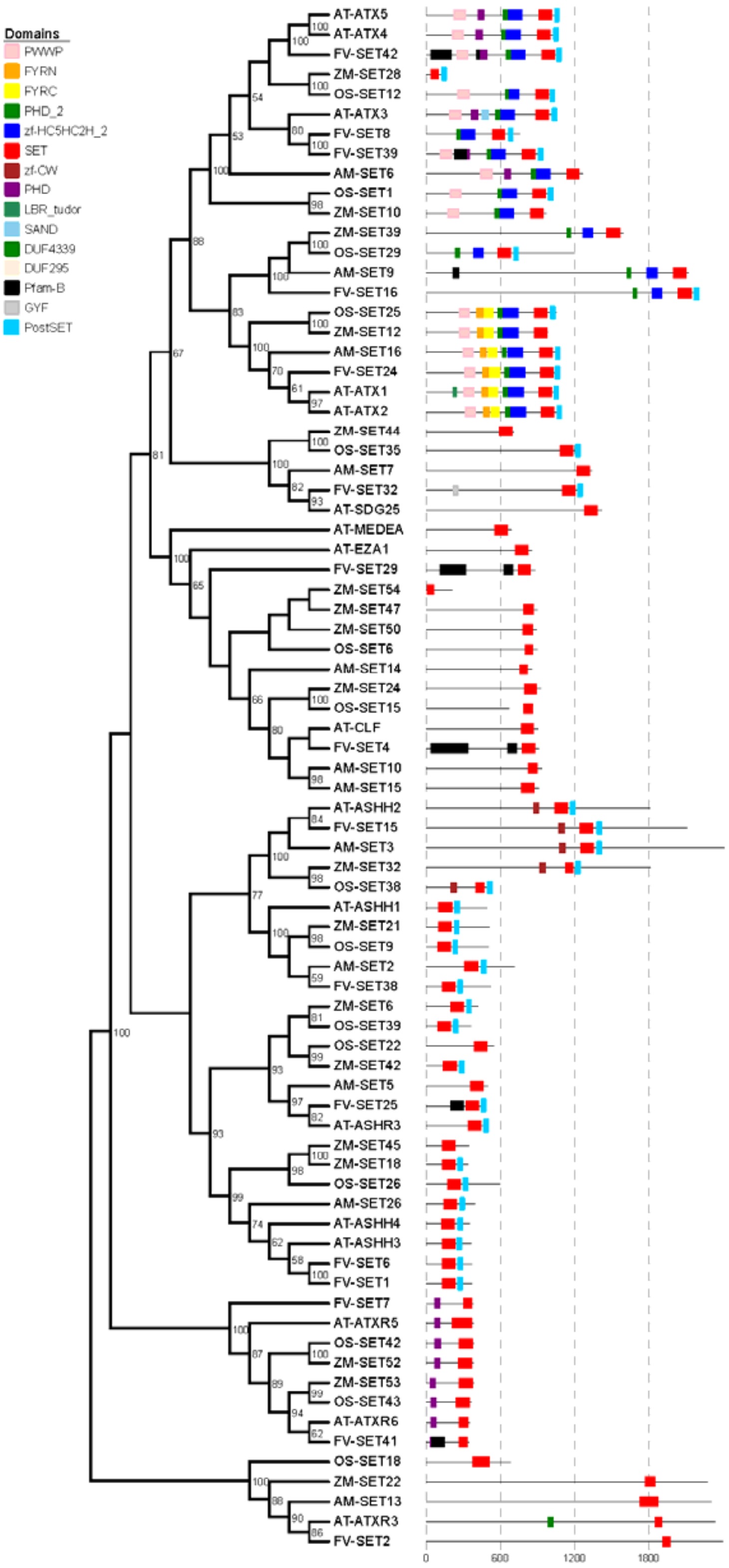

Supplementary Figure S4. A most likelihood phylogenetic tree and domain compositions of class V SET genes identified in *F. vesca*, *A. thaliana*, *O. sativa*, *Z. mays* and *A. trichopod*). The phylogenetic tree was constructed based on the amino acids sequences of the whole proteins with 1000 bootstrapping replicates, and the results of the bootstrapping analysis larger than 50% are shown.

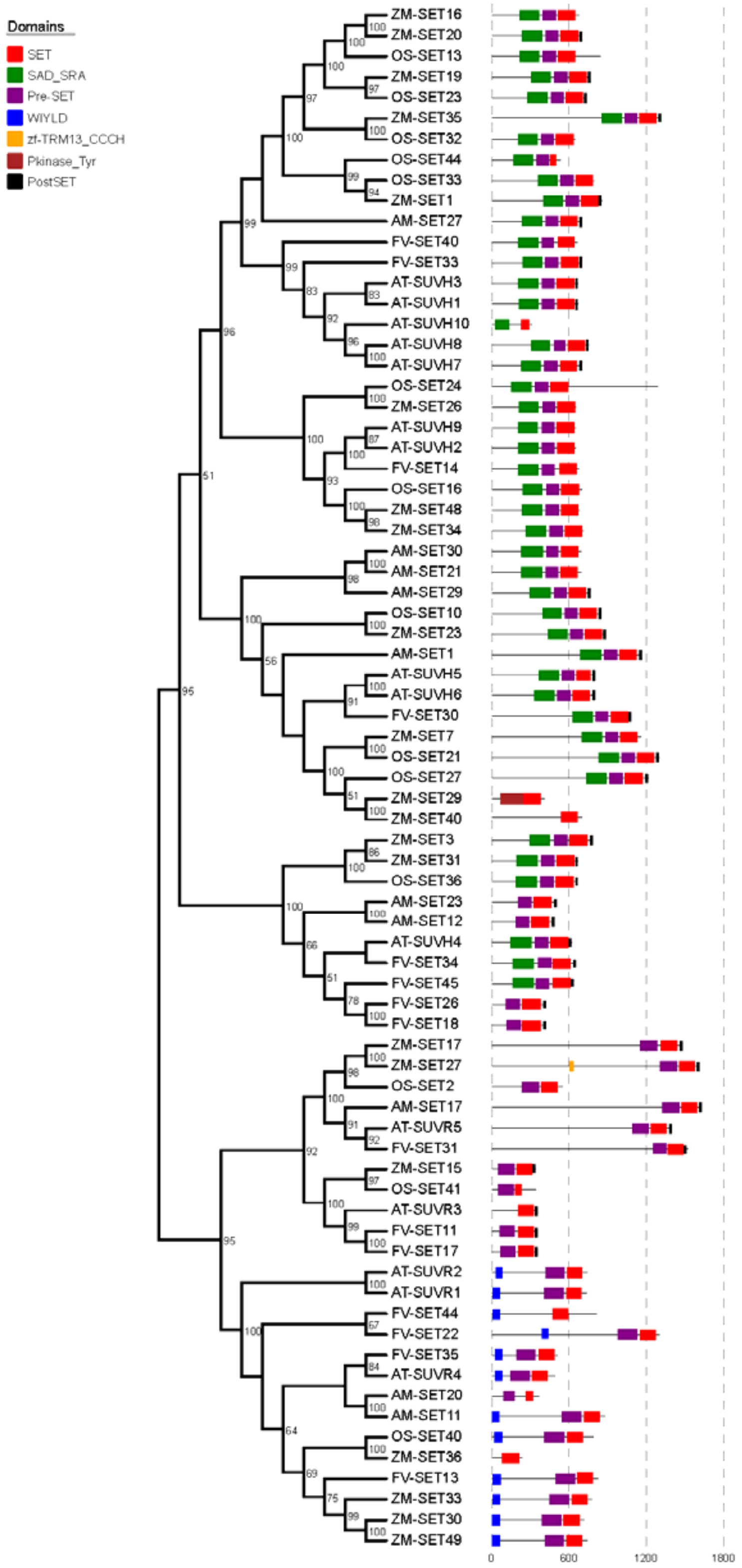

Supplementary Figure S5 A most likelihood phylogenetic tree and domain compositions of class VI SET genes identified in *F. vesca*, *A. thaliana*, *O. sativa*, *Z. mays* and *A. trichopod*). The phylogenetic tree was constructed based on the amino acids sequences of the whole proteins with 1000 bootstrapping replicates, and the results of the bootstrapping analysis larger than 50% are shown.

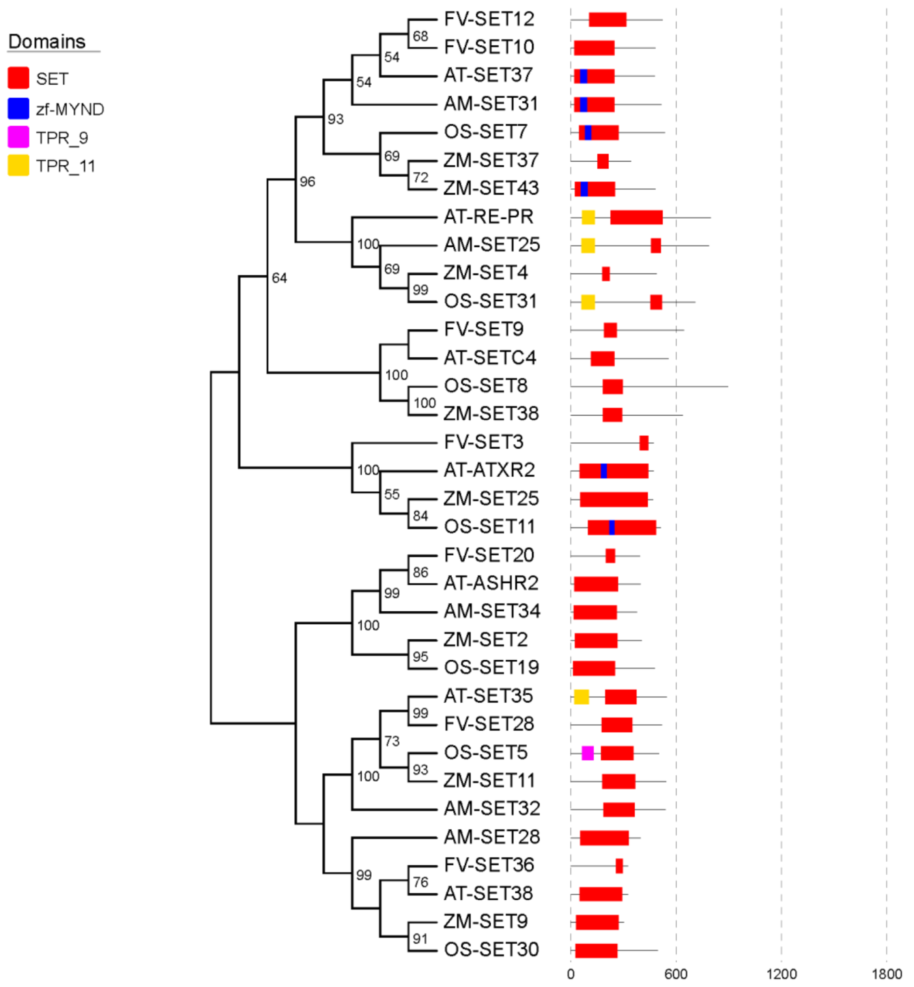

Supplementary Figure S6. A most likelihood phylogenetic tree and domain compositions of class VII SET genes identified in *F. vesca*, *A. thaliana*, *O. sativa*, *Z. mays* and *A. trichopod*). The phylogenetic tree was constructed based on the amino acids sequences of the whole proteins with 1000 bootstrapping replicates, and the results of the bootstrapping analysis larger than 50% are shown.

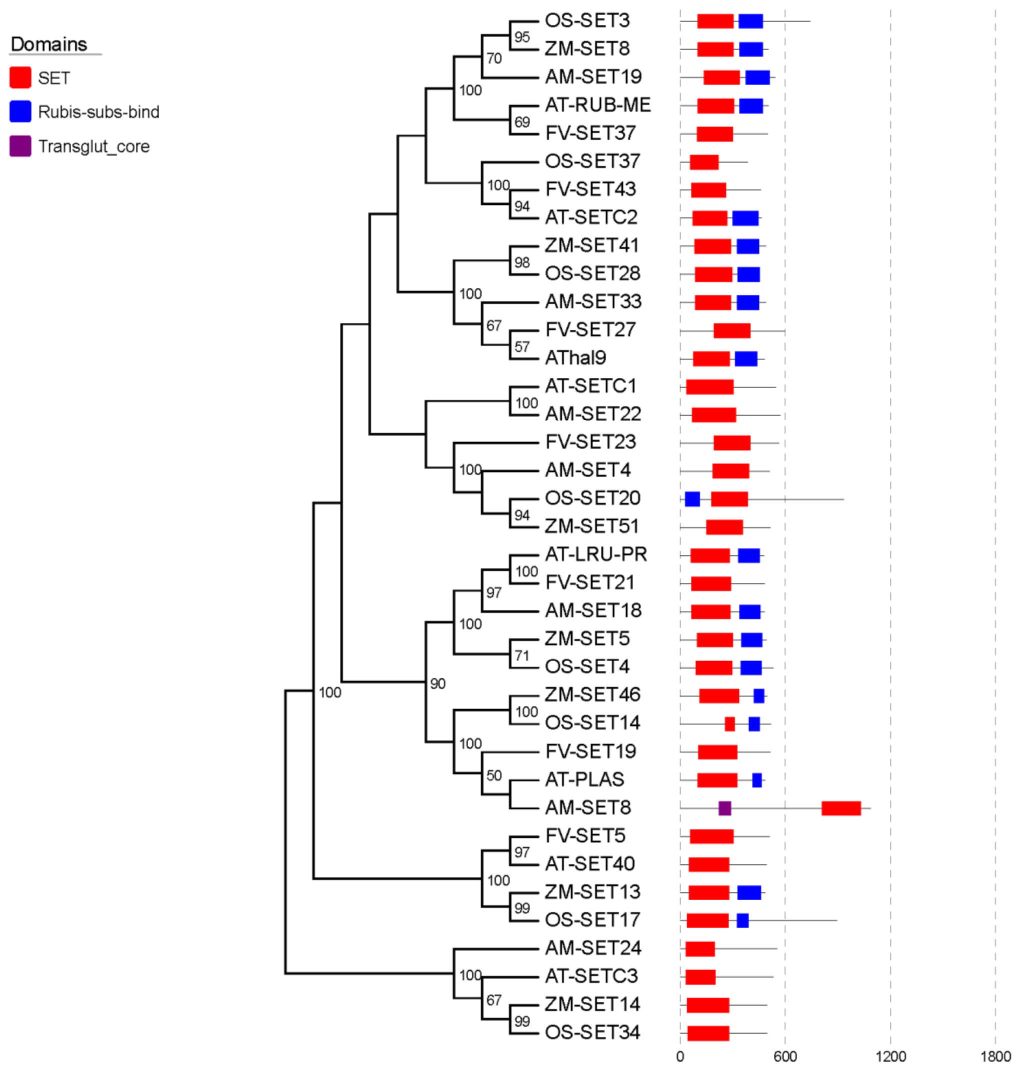

Supplementary Figure S7. A most likelihood phylogenetic tree and domain compositions of SET genes identified in *F. vesca*, *A. thaliana*, *V. vinifera, N. nucifera*, *O. sativa*, *Z. mays*, *A. trichopoda* and *S. moellendorffii*). The phylogenetic tree was constructed based on the amino acids sequences of SET domains with 1000 bootstrapping replicates, and the results of the bootstrapping analysis larger than 50% are shown. The class IV SET genes are marked as red.

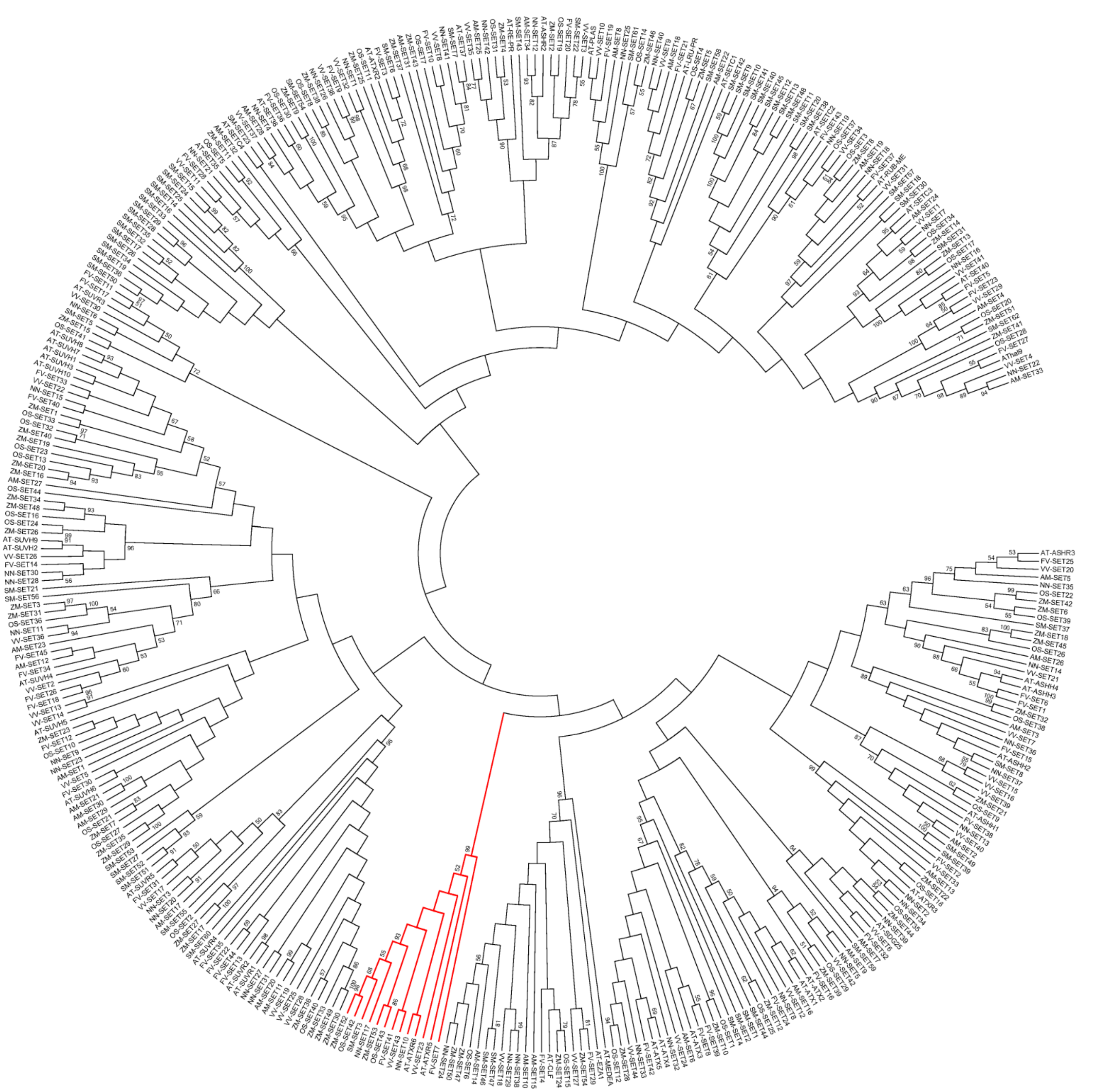

Supplementary Figure S8. A most likelihood phylogenetic tree domain composition and exon/intron construction of the predicted JmjC genes identified in the five species. They phylogenetic tree is shown in Fig. 4A as well.

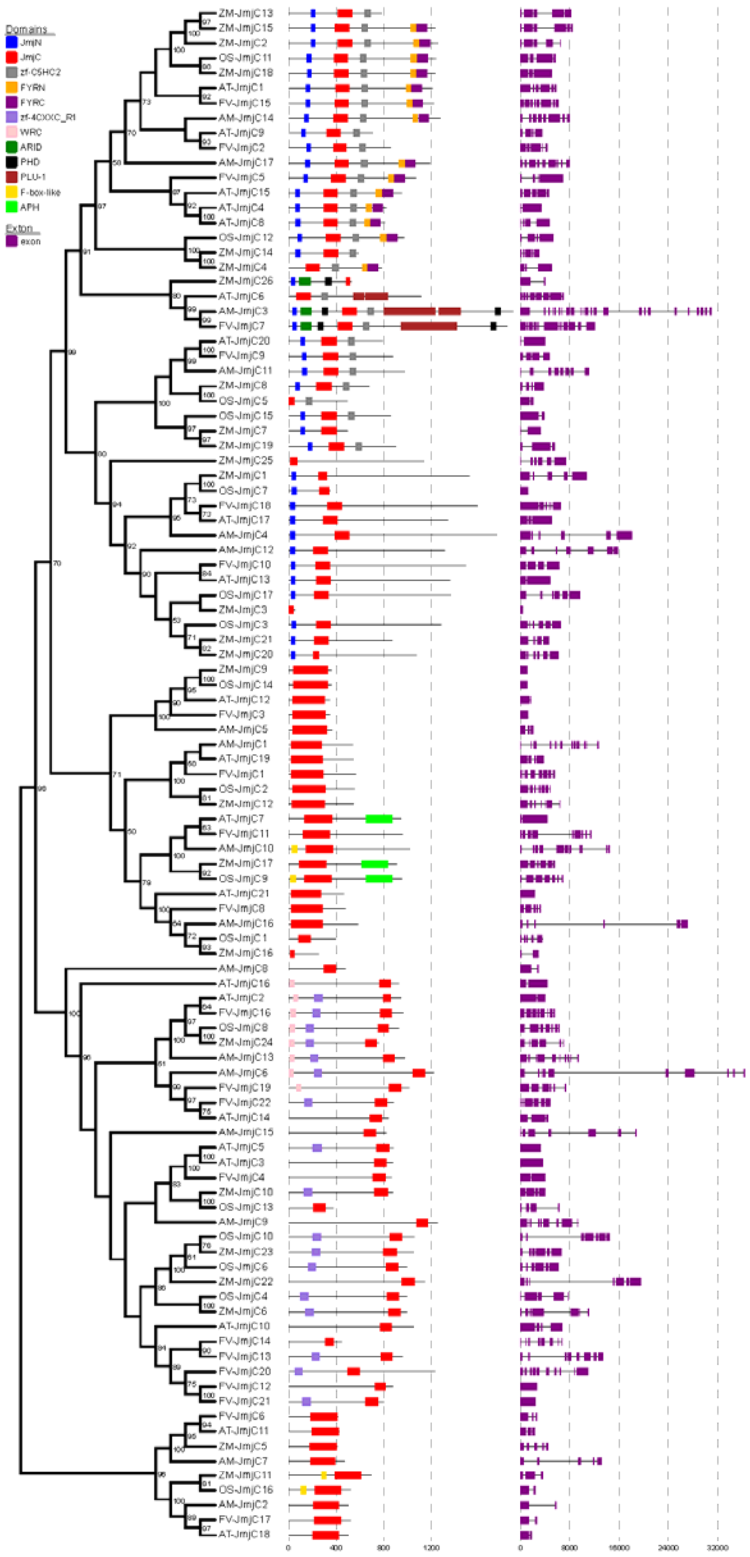

Supplementary Figure S9. A most likelihood phylogenetic tree domain composition and exon/intron construction of the predicted LSD genes identified in the eight species. They phylogenetic tree is shown in Fig. 4B as well.

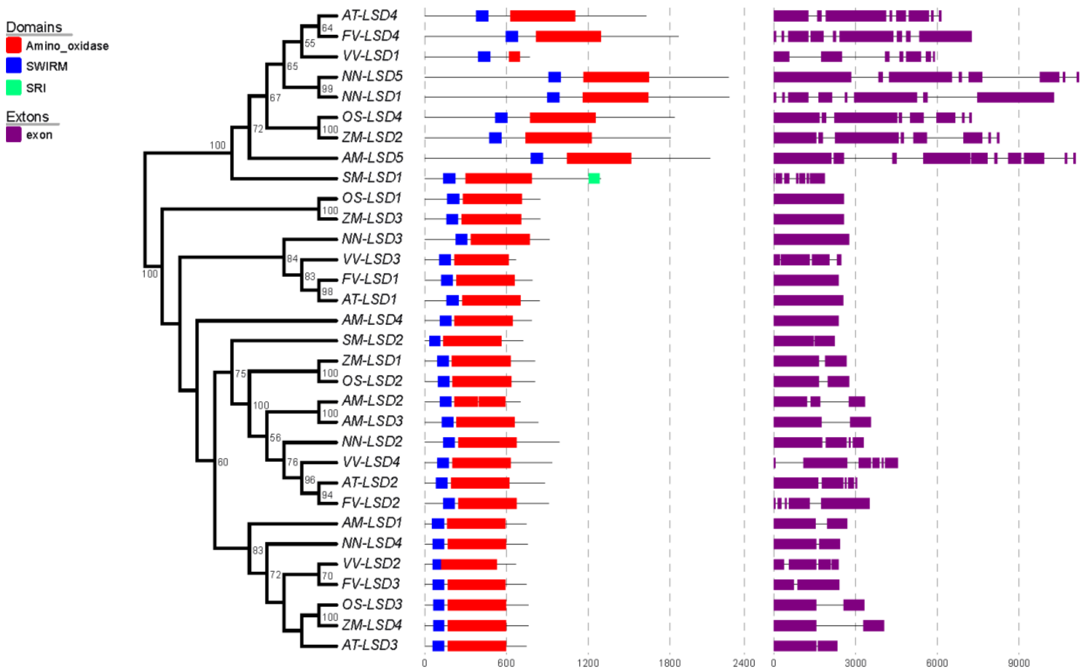

Supplement: Supplementary Information [file srep23581-s1.pdf]
